# Retrospective Longitudinal Radiographic Evaluation of Non-Surgically Managed Jaw Lesions Using Panoramic Radiography

**DOI:** 10.3390/medicina62010034

**Published:** 2025-12-24

**Authors:** Tuna Sumer, Ayşe Pınar Sumer

**Affiliations:** 1 Department of Oral and Maxillofacial Surgery, Faculty of Dentistry, University of Ondokuz Mayıs, 55270 Samsun, Turkey; 2 Department of Dentomaxillofacial Radiology, Faculty of Dentistry, University of Ondokuz Mayıs, 55270 Samsun, Turkey; psumer@omu.edu.tr

**Keywords:** panoramic radiograph, jaw lesions, radicular cyst

## Abstract

*Background and Objectives*: The aim of this study was to evaluate the radiographic progression of non-surgically managed jaw lesions that remained untreated due to patient deferral or refusal of surgery. Radiographic changes were assessed using two panoramic radiographs obtained at different time points, with a focus on dimensional progression, morphological characteristics, and anatomical involvement. *Materials and Methods*: A total of 85 non-surgically managed intraosseous cystic and cyst-like jaw lesions were evaluated on two panoramic radiographs obtained at least one year apart. Histopathological confirmation was available for 26 of the lesions (30.6%), while the remaining cases were evaluated radiographically due to the absence of surgical intervention or accessible pathology records. Assessments included localization, size, shape, internal structure, borders, association with non-erupted teeth, root resorption, tooth displacement, involvement of anatomical structures, and cortical changes such as thinning, expansion, or destruction. Nonparametric statistical comparisons were used to assess time-dependent changes and differences between follow-up groups. *Results*: A total of 57 lesions occurred in the mandible and 28 in the maxilla, predominantly in the posterior regions. The mean vertical/horizontal measurements of the intraosseous lesions was found to be 10.9 ± 4.6 mm/12.2 ± 6.5 mm (Mean ± SD) on the initial panoramic radiographs (Med: 10.0–IQR: 6.50/Med: 12.0–IQR: 8.75) and 14.8 ± 5.3 mm/17.5 ± 8.3 mm (Mean ± SD) on the second panoramic radiographs (Med: 14.5–IQR: 6.75/Med: 16.0–IQR: 10.75), respectively. Both vertical and horizontal dimensions showed a statistically significant increase between the two time points (*p* < 0.05). Initially, 41 lesions exhibited corticated margins; at follow-up, an additional 33 non-corticated lesions developed cortication. Lesions without corticated margins on the initial images exhibited significantly greater vertical and horizontal growth than those with corticated borders (*p* < 0.05). Lesions followed for 3–5 years showed significantly greater dimensional changes compared with those observed for shorter or longer intervals (*p* < 0.05). Lesion shape, internal structure, and multilocularity remained largely stable. *Conclusions*: Within the limitations of this retrospective study, non-surgically managed jaw lesions showed a tendency to increase in size over time. While the development of corticated borders may be associated with reduced growth activity, panoramic radiography alone is insufficient for definitive assessment, and regular radiographic follow-up should be considered within a broader clinical context.

## 1. Introduction

The jaws exhibit several distinct characteristics that differentiate them from other bones in the human skeleton. Embryologically, they are primarily derived from cranial neural crest cells, referred to as ectomesenchyme rather than solely from mesodermal tissue. One of the most unique features of the jaws is the presence of teeth, which gives rise to a wide variety of odontogenic cysts and tumors [1]. As a result, intraosseous jaw lesions represent a common diagnostic entity in dental and maxillofacial radiology and are frequently encountered during routine imaging [2].

Many jaw lesions are surgically treated to prevent complications such as expansion, infection, or pathological fracture and it is essential that such lesions are detected as early as possible to minimize any necessary surgery [3]. However, not all patients undergo surgical management. Previous studies have shown that some individuals delay or decline dental and surgical treatment for reasons such as the absence of symptoms, financial constraints, dental anxiety, fear and phobia, limited time, and personal treatment preferences [4,5]. As a result, these lesions may remain untreated for extended periods, providing an opportunity to observe their radiographic progression and presumed natural course.

Panoramic radiography is a widely used imaging modality that provides a comprehensive view of the maxillofacial region and plays a vital role in initial diagnosis and follow-up evaluations. Although it has limitations compared to cone-beam computed tomography (CBCT), such as lower spatial resolution and the inability to provide cross-sectional views, its advantages in terms of availability, cost-effectiveness, and lower radiation dose make it particularly suitable for long-term follow-up studies. In panoramic images, radiographic features such as lesion localization or distribution, margin definition, internal structure, and the effect on and relationship with adjacent anatomical structures provide valuable diagnostic information [6,7]. However, despite the widespread use of panoramic radiography, there is limited longitudinal evidence focusing specifically on the natural radiographic course of non-surgically managed jaw lesions. The present study is novel in that it provides a long-term, retrospective evaluation of dimensional and morphological changes in untreated jaw lesions using panoramic radiographs (PANs).

The relative frequency of intraosseous jaw lesions varies across populations due to demographic, cultural, ethnic, and geographical differences, as demonstrated in multiple epidemiological studies [3,8,9,10,11]. Studies have consistently reported odontogenic cysts as the most common category of intraosseous jaw lesions, with radicular cysts being the most frequently encountered subtype [12,13]. Although odontogenic cysts may occur across a broad age range, radicular cysts are most frequently diagnosed between the third and sixth decades of life, while dentigerous cysts are commonly observed in patients aged 10–30 years. Approximately 60% of odontogenic keratocysts are reported in individuals between 10 and 40 years of age. Overall, these cyst types exhibit a slight male predominance [6,7]. To date, the long-term radiographic behavior of non-surgically managed jaw lesions remains poorly documented. There is no clear consensus regarding whether such lesions remain stable, enlarge progressively, or regress spontaneously over time. While some lesions appear radiographically unchanged for extended periods, others may demonstrate substantial increases in size or changes in morphology. The demographic characteristics of the present study population provide a relevant context for evaluating lesion behavior in patients who commonly delay or defer treatment.

This retrospective longitudinal study aims to evaluate changes in lesion size and morphology in patients with jaw lesions who did not undergo surgical treatment. PANs taken at two different time points, with a minimum interval of one year, were analyzed to assess the radiographic evolution of these lesions over time. Gaining insight into the natural progression of untreated jaw lesions may enhance clinicians’ understanding of their behavior and support evidence-based decision-making regarding follow-up intervals and treatment planning. Moreover, increasing patient awareness about the potential for lesion progression may encourage regular monitoring and reduce the likelihood of neglect due to the absence of clinical symptoms.

## 2. Materials and Methods

### 2.1. Study Design and Sample Selection

The present study was designed as a retrospective analysis of patients who had previously been diagnosed with jaw lesions and had undergone radiographic follow-up without surgical intervention. Patient records from the Department of Dentomaxillofacial Radiology were reviewed to identify individuals who had non-surgically managed intraosseous jaw lesions and at least two PANs obtained at different time points with a minimum interval of one year between them.

A total of 83 patient records meeting these criteria were identified and included in the study. Histopathological confirmation was available for 26 of the lesions. In the remaining cases, histopathological data were unavailable due to patient refusal or postponement of surgical intervention, loss to follow-up, or inability to re-contact patients, or treatment having been performed at external or private institutions without accessible pathology records. Accordingly, these lesions were assessed solely on the basis of their radiographic features and were not assigned definitive diagnostic labels unless histopathological confirmation was available.

Overall, the lesions analyzed encompassed a spectrum of cystic and cyst-like intraosseous entities commonly encountered in dental and maxillofacial radiology, and the primary focus of the study was on their longitudinal radiographic evolution rather than on diagnostic classification.

The study protocol was approved by the University Clinical Research Ethic Committee.

Inclusion criteria: availability of at least two PANs taken at different time points with a minimum interval of one year; radiographically identifiable jaw lesions; no documentation of surgical or other interventional treatment during the follow-up period.

Exclusion criteria: records showing previous surgical intervention or endodontic treatment related to the lesion; radiographs with insufficient diagnostic quality; patients with systemic disorders potentially affecting bone metabolism or lesion behavior (e.g., bone metabolic disorders, systemic malignancies).

### 2.2. Radiographic Evaluation

PANs were analyzed for changes in lesion characteristics based on the criteria outlined by Lim et al. [9]. Each lesion was evaluated on two PANs taken at different time intervals to assess the following radiographic features: lesion shape (round/ovoid-scalloped-irregular), border definition, border cortication, cortication continuity, internal contents, presence of multilocular appearance, impact on anatomical structures, expansion of surrounding anatomic boundaries, cortical thinning, cortical destruction, tooth displacement and root resorption. Given the inherent limitations of panoramic radiography, certain features such as expansion of surrounding anatomic boundaries, cortical thinning and continuity of cortication were interpreted with caution and considered semi-quantitative rather than definitive assessments.

Additionally, lesion dimension was measured in both vertical and horizontal planes on PANs to assess changes in size over time. Measurements were performed using calibrated digital radiographic software, which automatically applies scale correction based on imaging parameters (TurcaSoft HBYS, version 1.1.0, license No: 11550020). These measurements were limited to lesions with a round/ovoid and well-defined shape; lesions with irregular or scalloped borders were excluded from dimensional analysis due to the difficulty in obtaining reliable and reproducible measurements in such cases. The maximum vertical dimension (in mm) of the area was recorded as vertical measurement and the maximum dimension (in mm) perpendicular to the previously obtained vertical measurement was recorded as horizontal measurement [14]. All measurements were performed by a single dentomaxillofacial radiologist with 30 years of experience. In line with the study design, inter-observer agreement was not assessed; instead, intra-observer reliability was evaluated through repeated measurements after a two-week interval, yielding excellent ICC values.

### 2.3. Statistical Analysis

Intra-observer reliability was assessed using intra-class correlation coefficients (ICC) based on a one-way random-effects model to evaluate agreement between repeated measurements obtained by the same observer. The ICCs for the measurements ranged from 0.943 to 0.994; an ICC of ≥0.9 was considered satisfactory [15].

This study is a retrospective observational study that investigates changes between two time points. The data examined in the study consist of lesion diameters (vertical and horizontal dimensions) and their percent growth as a measure of change over time. The normality assumptions were assessed using the Shapiro–Wilk test, and it was determined that the data did not follow a normal distribution; therefore, nonparametric approaches were employed for analysis. The time-related changes in continuous variables were compared across three groups: follow-up intervals of 1–2 years, 3–4 years, and >5 years. Kruskal–Wallis H test was used for these group comparisons, and when significant differences were found, Dunn’s test was applied for multiple pairwise comparisons. Growth percentage was defined as [(follow-up − baseline)/baseline measurement] × 100, and differences between two independent groups were assessed with the Mann–Whitney U test. Differences between the first and second PANs for vertical and horizontal measurements were determined using the Wilcoxon test due to dependence. To describe the direction and magnitude of the changes, means, standard deviations (SD), median (med), interquartile range (IQR), minimum and maximum values were reported. Additionally, to evaluate agreement for categorical attributes across the two time points, the Kappa (κ) statistic was calculated, and κ values were interpreted according to the Strength of Agreement scale. All tests were two-tailed, with *p*-values less than 0.05 considered statistically significant. Analyses were conducted using the SPSS version 21 package.

## 3. Results

A total of 83 patients with intraosseous jaw lesions were included in the study. PANs taken at two different time points were available for each patient, with the interval between the first and the last radiograph ranging from a minimum of 1 year to a maximum of 13 years (mean 4 years). The sample consisted of 31 females and 52 males, with a mean age of 36 years. In 2 patients, two separate lesions were identified, resulting in a total of 85 lesions evaluated.

Of these lesions, 57 were located in the mandible and 28 in the maxilla. Regional distribution showed that 61 lesions were situated in the posterior jaw regions and 24 in the anterior areas. A total of 26 lesions were associated with an impacted tooth, of which 23 were related to mandibular third molars, two to maxillary canines, and one to a mandibular canine. Histopathological diagnoses were available for 26 lesions. Among these, 17 were identified as radicular cysts, three as odontogenic keratocysts, and six as dentigerous cysts.

Out of 85 lesions, 68 exhibited a relatively well-defined oval shape, allowing for dimensional measurements. The horizontal dimension of the lesions was statistically significantly greater than the vertical dimension in both radiographs (*p* < 0.05).

**Figure 1:**
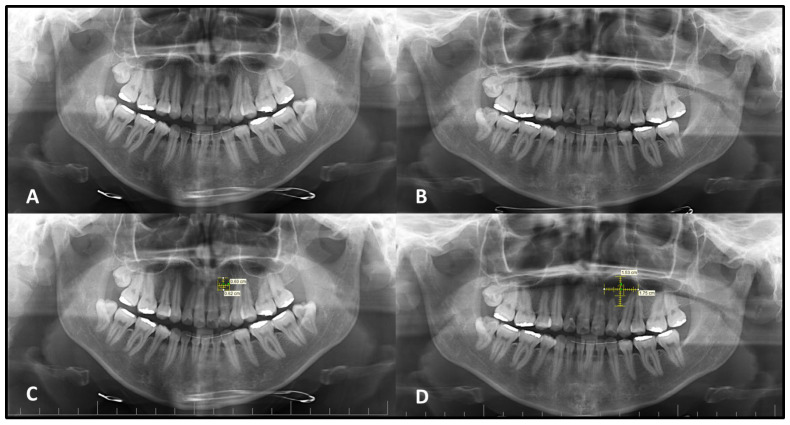
An oval, well-defined radiolucent lesion located at the apex of the maxillary lateral incisor is seen on the initial panoramic radiograph (**A**). Three years later, the lesion had increased in size and developed corticated margins (**B**). Horizontal and vertical dimensions of the lesion are illustrated on the initial (**C**) and second (**D**) radiographs.

The mean vertical measurement of the intraosseous lesions was found to be 10.9 ± 4.6 mm (Mean ± SD) on the initial PANs (Med: 10.0–IQR: 6.50) and 14.8 ± 5.3 mm (Mean ± SD) on the second PANs (Med: 14.5–IQR: 6.75). A statistically significant increase was observed in vertical dimensions between the two time points (*p* < 0.05). For horizontal measurements, the mean values were 12.2 ± 6.5 mm (Mean ± SD) on the initial images (Med: 12.0–IQR: 8.75) and 17.5 ± 8.3 mm on the second PANs (Med: 16.0–IQR: 10.75). This difference was also found to be statistically significant (*p* < 0.05), indicating progressive enlargement in both vertical and horizontal dimensions over time (Figure 1). Because follow-up intervals varied widely (1–13 years), lesions were analyzed in predefined interval groups (1–2 years, 3–4 years, and >5 years) to minimize the effect of heterogeneity on comparability. Lesions with a follow-up interval of 3–5 years demonstrated statistically significantly greater dimensional changes in both horizontal and vertical planes compared to those monitored for 1–2 years or more than 5 years (*p* < 0.05) (Table 1 and Table 2).

In the evaluation of lesion margins, a distinct pattern of cortication was observed over time. On the initial PANs, 76 out of 85 lesions exhibited well-defined borders, of which 41 showed corticated margins and 17 presented with continuously corticated borders. By the time of the second radiograph, 33 of the 44 lesions that were non-corticated in the first radiograph had developed corticated borders. Additionally, 16 of the 68 lesions exhibited continuously corticated margins on the second image. Most of the radicular cysts followed this pattern of increasing cortication over time. Among 17 radicular cysts, 15 exhibited well-defined borders on the initial PAN. Of these, 9 showed no cortication initially; however, 8 developed corticated margins on the second radiograph. However, all three odontogenic keratocysts and five of the six dentigerous cysts demonstrated consistently well-defined and corticated borders on both the initial and second PANs.

**Figure 2:**
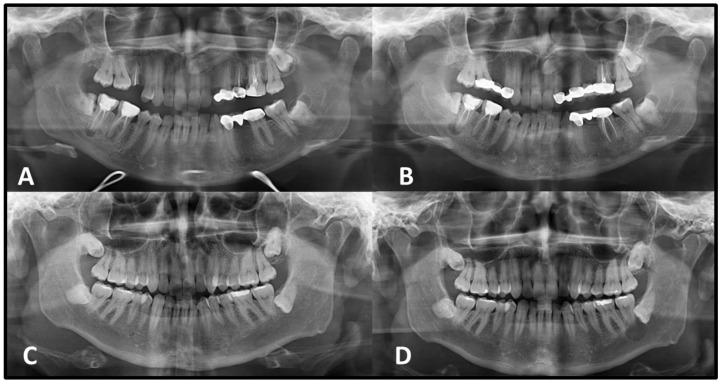
A unilocular radiolucent lesion with well-defined corticated margins associated with an impacted left mandibular third molar is seen on the initial panoramic radiograph (**A**). The lesion appeared radiographically stable with no significant changes two years later (**B**). A radicular cyst with well-defined corticated margins associated with an impacted left mandibular third molar is seen on the initial panoramic radiograph (**C**). After a 5-year interval, the lesion exhibited mild enlargement and extended into the inferior alveolar canal (**D**).

**Figure 3:**
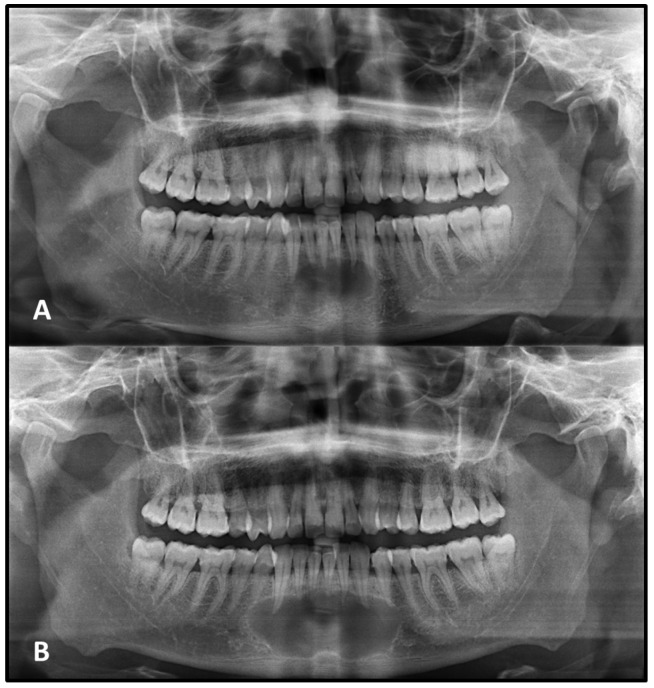
A radicular cyst located in the anterior mandible with well-defined but non-corticated borders is seen on the initial panoramic radiograph (**A**). After two and a half years, the lesion showed considerable enlargement and developed continuous corticated margins (**B**).

Statistically significant differences were observed in both vertical and horizontal dimensional changes between lesions with corticated and non-corticated borders on the initial PANs (*p* < 0.05). Lesions lacking corticated margins demonstrated a greater percentage of growth over time compared to those with corticated boundaries (Table 3, Figure 2 and Figure 3).

Regarding lesion shape, internal structure, and multilocular appearance, no consistent radiographic changes were observed between the first and second PANs. These features appeared stable over time in the majority of cases. All lesions were radiolucent on both PANs, except for one with a mixed appearance. Two lesions exhibited a multilocular configuration at both time points, whereas an additional two lesions that were initially unilocular on the first radiograph demonstrated a multilocular appearance on the follow-up image.

Findings related to the expansion of surrounding anatomical boundaries, cortical thinning, and cortical destruction were limited due to the inherent resolution constraints of panoramic radiography. In the initial PANs, expansion, cortical thinning, and resorption were observed in 15, 9, and 6 lesions, respectively. In the second radiograph, an additional 16 expansions, 12 cortical thinning, and 9 resorptions were identified, indicating a notable increase in these findings over time.

**Figure 4:**
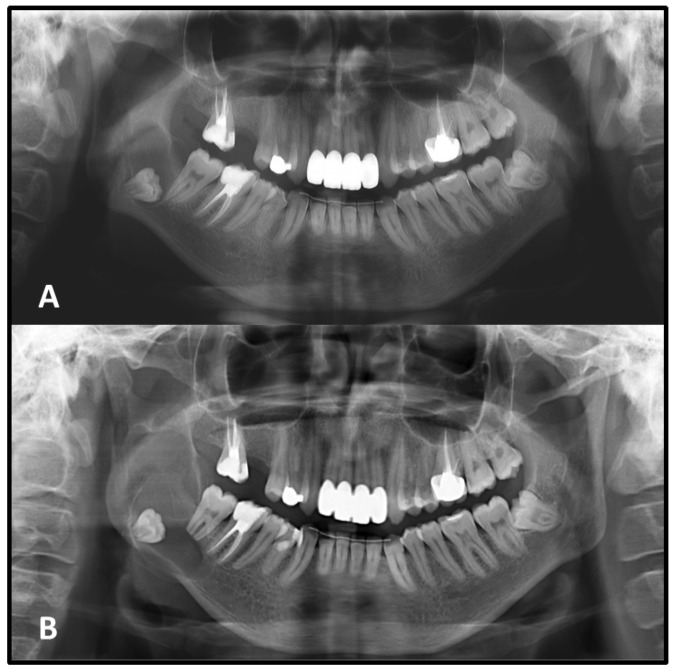
A unilocular radiolucent lesion with well-defined borders associated with an impacted right mandibular third molar observed on the initial panoramic radiograph. The lesion demonstrates mild cortical expansion, is in proximity to the inferior alveolar canal, and has caused displacement of the impacted tooth (**A**) The same lesion demonstrated considerable growth over the three-year interval, with displacement of the impacted third molar toward the inferior mandibular border, extension into the inferior alveolar canal, and associated root resorption of the adjacent second molar. Marked cortical expansion, thinning, and cortical destruction were also observed (**B**).

Tooth root resorption associated with the lesion was identified in 9 cases on the initial PAN. Among these, resorption remained stable in 7 cases, while 1 case showed progression, and another case exhibited involvement of additional roots at follow-up. In the second image, root resorption was newly detected in 8 additional cases that had shown no evidence of resorption initially (Figure 4).

Tooth displacement was noted in 3 cases on the initial PAN and remained stable at follow-up. In an additional 3 cases, displacement of adjacent teeth was newly detected on the second radiograph, despite no such findings on the initial image.

**Figure 5:**
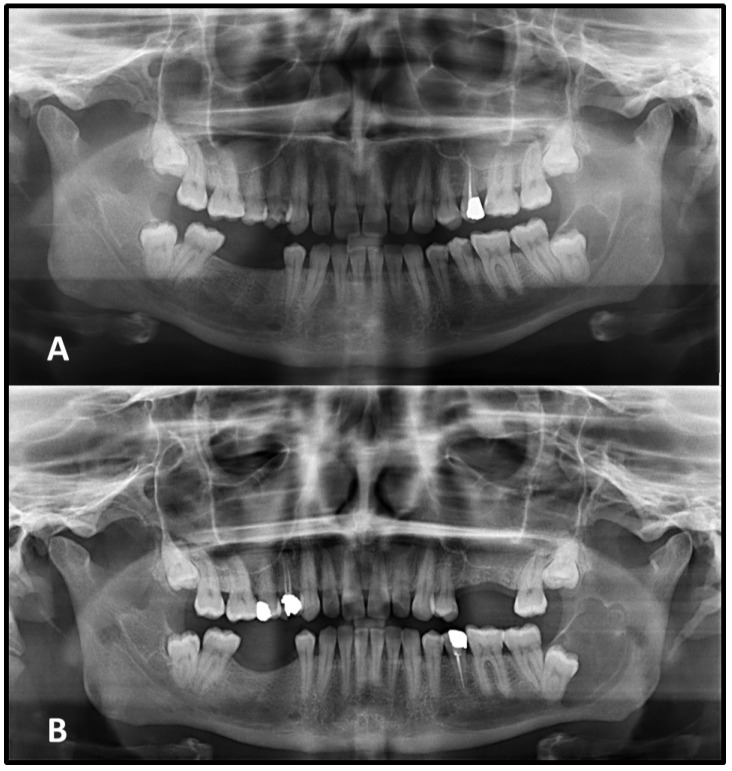
A odontogenic keratocyst with well-defined corticated borders associated with an impacted left mandibular third molar is evident on the initial panoramic radiograph (**A**). After three years, the lesion had increased in size, causing displacement of the impacted tooth and extending into the inferior alveolar canal (**B**).

Changes in the relationship between the lesion and adjacent anatomical structures were observed during follow-up. Seventeen lesions that initially showed no proximity to the IAC and 2 lesions unrelated to the IC on the first radiograph were later found to exhibit radiographic contact with these critical neurovascular structures in the second image, a clear radiographic association with these anatomical structures was observed. This shift suggests a possible extension of the lesion toward critical neurovascular pathways over time (Figure 5).

The overall agreement between first and second panoramic imaging in regard to lesion characteristics is shown in Table 4. Strong agreements between initial and second PANs were seen in lesion’s shape, internal contents, multilocularity and causing tooth displacement. Weak agreements were seen in the presence of well-defined lesion borders and border cortication. Additionally, moderate agreement was observed between initial and second PANs in continuity of border cortication, affecting the IC/IAC, expansion, cortical thinning, destruction and causing tooth displacement.

## 4. Discussion

The patient’s perception of lesion duration plays a critical role in clinical assessment. Lesions that have persisted over long periods are typically suggestive of developmental or benign pathologies, whereas those that emerge and progress rapidly are more often associated with reactive, infectious, or malignant processes [6,7]. However, the accuracy of a patient’s reported medical history may be limited due to factors such as inattention, cognitive impairment, or denial of illness, making it challenging to determine the true onset and progression of the lesion. In asymptomatic cases, patients may underestimate the clinical significance of the lesion, delay seeking care due to fear or uncertainty, or intentionally postpone evaluation. Such delays may contribute to dimensional and morphological changes in the lesion, ultimately complicating both diagnosis and treatment planning [6]. Lesions such as the odontogenic keratocyst can grow to a large size, resulting in facial deformity, destruction of surrounding structures and difficult surgical management [3]. In the present study, 83 patients who had deferred surgical treatment for various reasons were evaluated, and the sample demonstrated a clear male predominance. This distribution is consistent with epidemiological reports indicating a slight male predilection for several odontogenic cyst types [6,7]. In a Turkish population, Kılınc et al. [17] reported a male-to-female ratio of 1.7:1 for odontogenic and non-odontogenic cysts. Similarly, Almazyad et al. [12] found that 60.4% of cases occurred in males. It is also possible that behavioral or socioeconomic factors may contribute to treatment delay among male patients; however, the retrospective nature of the study does not allow for direct assessment of such influences. Therefore, the observed male predominance should be interpreted descriptively and within the context of known epidemiological trends.

Lesion size can have diagnostic implications, particularly when considered alongside the estimated duration of the lesion, as this combination may provide an approximate rate of growth or enlargement [6]. In addition, when cysts reach large sizes, they can alter the biomechanical properties of the jaws and weaken structural integrity [18]. In the present study, radiographic comparison between two time points revealed that many lesions exhibited dimensional increases over time, particularly in the horizontal plane. In lesions with irregular or scalloped borders, quantitative measurement was not performed due to difficulties in obtaining reproducible values. However, qualitative radiographic evaluation suggested that these lesions also showed a tendency to enlarge over time. This progressive enlargement, even in the absence of clinical symptoms, may reflect ongoing biological activity and reinforces the need for radiographic monitoring in cases where surgical treatment is delayed or deferred.

Cysts that originate within bone typically exhibit well-defined corticated margins, often represented by a thin radiopaque line; however, secondary infection can alter this appearance, rendering the borders thicker, sclerotic, or poorly defined. Generally, lesions with well-defined margins are considered benign, while ill-defined margins tend to suggest more aggressive, inflammatory, or neoplastic processes [7]. A radiographic pattern observed in this study was the progressive cortication of lesion margins over time, particularly among lesions that initially presented without corticated borders. This trend was most frequently observed in radicular cysts, many of which developed corticated margins during follow-up. Lesions with corticated borders on the initial PAN tended to show smaller dimensional changes, whereas those lacking cortication at baseline generally exhibited greater enlargement and more frequent development of partial or complete cortication over time. These radiographic patterns suggest that the presence or absence of cortication may be associated with different trajectories of radiographic change; however, these observations reflect radiographic changes only; no causal or biological interpretations were inferred from these findings. Additionally, changes in the appearance of cortication on PANs may be influenced not only by biological processes but also by technical factors such as projection differences, unequal magnification and geometric distortion, patient positioning, and measurement variability inherent to two-dimensional imaging [7].

Although previous studies have suggested that intracystic pressure may contribute to early expansion of some cystic lesions, this mechanism remains incompletely understood and could not be evaluated in the present study due to the retrospective design and the reliance on panoramic radiography [9,19,20]. Odontogenic keratocysts, although limited in number and not subjected to statistical analysis, demonstrated consistently well-defined corticated borders on both PANs, yet still exhibited measurable enlargement. This observation aligns with their known radiographic behavior and highlights that cortication does not necessarily indicate growth cessation [6,7]. Overall, these findings emphasize that changes in cortication patterns should be considered descriptive radiographic observations rather than indicators of specific biological processes, and they underline the importance of cautious interpretation when integrating radiographic progression into clinical decision-making.

In this study, lesions within the 3–5 year follow-up group showed greater dimensional change compared with the other intervals; however, this pattern likely reflects the distribution of follow-up durations in our sample rather than a biologically meaningful phase. The limited growth observed in short-term cases may simply reflect the brevity of observation, whereas some long-term cases may represent lesions that had already reached a more quiescent radiographic stage. Importantly, the study design does not allow identification of an optimal or characteristic timeframe for lesion progression, and these findings should therefore be interpreted with caution. The wide variability in follow-up intervals represents an inherent limitation of retrospective studies. Although stratification into predefined time-interval groups was used to improve comparability, heterogeneity in follow-up duration may still influence the observed growth patterns. Regular radiographic monitoring remains advisable, particularly when lesions demonstrate ongoing change.

In the present study, lesion shape, internal structure, and the presence of multilocular or unilocular patterns showed minimal radiographic change between the initial and subsequent PANs, suggesting that these features remained morphologically stable over time regardless of dimensional growth. The vast majority of lesions remained radiolucent and unilocular throughout the observation period, which is consistent with the typical appearance of slow-growing benign jaw lesions [7]. However, in three cases, lesions that initially appeared irregular in shape exhibited a transformation into an ovoid configuration over time, possibly reflecting a tendency toward maturation or encapsulation. Additionally, two lesions that were unilocular on the initial radiograph demonstrated a multilocular pattern at follow-up, indicating that although uncommon, structural changes can occur. Notably, no changes in radiolucency were observed in any of the cases, further supporting the generally indolent nature of these lesions.

Cysts may cause tooth displacement and root resorption when they reach large sizes. The roots of teeth may be resorbed by either benign or malignant lesions, but root resorption is more commonly associated with benign processes [7]. It is often associated with prolonged pressure from adjacent expanding lesions and may serve as an important radiographic indicator of lesion behavior [7,21,22,23]. In the present study, a limited number of non-surgically managed lesions exhibited root resorption that was absent or minimal on the initial PAN but became apparent or progressed in the subsequent image. However, the interpretation of root resorption on PANs is limited by their two-dimensional nature and subtle changes may therefore be underestimated compared with CBCT. Displacement of teeth is also seen more commonly with slower-growing, space-occupying lesions. A benign lesion exerts pressure on neighboring structures, resulting in the displacement of teeth or bony cortices [5]. In the present study, tooth displacement was observed even less frequently, generally in cases with significant lesion enlargement. This change may be attributed to the gradual thinning and remodeling of the adjacent bone, reducing mechanical resistance and facilitating positional shifts in neighboring teeth.

Cysts may displace the inferior alveolar nerve canal in an inferior direction or invaginate into the maxillary antrum [5]. In the present study, some lesions that initially had no radiographic contact with the inferior alveolar canal (IAC) in the mandible and the incisive canal (IC) in the maxilla were found to encroach upon these anatomical landmarks in the later radiographs. As lesion boundaries expand, they may gradually approximate or compress adjacent anatomical structures, potentially increasing the risk of neurosensory disturbances or complicating future surgical interventions [7]. These findings underscore the dynamic nature of seemingly indolent lesions and highlight the critical role of radiographic surveillance, not only for monitoring dimensional changes but also for evaluating evolving anatomical relationships that may influence both prognosis and treatment planning.

Odontogenic lesions can expand the mandible, usually in a smooth, curved manner, and change the buccal and lingual cortical plate into a thin cortical boundary [7]. They may also cause resorption and expansion of one or both cortical bones [21,22,23]. In the present study, radiographic features, including expansion of surrounding bone, cortical thinning, and cortical destruction, were observed in only a limited number of cases on PANs, and in some of these, such features became apparent only on the second image. This limited detection is likely attributable to the inherent imaging constraints of panoramic radiography, which lacks the spatial resolution and three-dimensional capability of CBCT. PAN views are curved image slices of the mandibulofacial tissues; there is less superimposition of structures and thus less of an interpretation problem, particularly for experienced clinicians [7]. CBCT provides superior assessment of intraosseous jaw lesions that cannot be reliably assessed on PANs [10]. The use of CBCT instead of PAN in the diagnosis of pathological or inflammatory maxillary sinus diseases contributes to a more accurate radiographic differential diagnosis [24]. CBCT also offers more accurate visualization of lesion–anatomy relationships and subtle cortical alterations, even when using low-dose protocols [25,26]. These findings highlight that while panoramic radiography remains useful for initial evaluation and long-term follow-up, CBCT is the preferred modality when precise three-dimensional assessment is required. Therefore, it is reasonable to assume that the actual prevalence of cortical changes may have been underestimated in this study.

This study has several limitations. The absence of histopathological confirmation for all cases limited the ability to correlate radiographic findings with specific lesion types. Additionally, the exclusive use of PANs may have led to limited detection of buccolingual changes, better visualized with CBCT. Variations in the time intervals between radiographs may also have influenced the consistency of the observed changes. Furthermore, the exclusion of irregularly shaped lesions from dimensional analysis may have introduced selection bias by underrepresenting lesions with more complex morphology.

## 5. Conclusions

In conclusion, the present retrospective analysis shows that non-surgically managed jaw lesions may exhibit dimensional and radiographic changes over time, with some lesions demonstrating alterations in cortication during follow-up. Lesions lacking corticated margins at baseline tended to show greater enlargement in this sample; however, these findings should be interpreted cautiously due to the methodological limitations of the study. The clinical significance of corticated versus non-corticated borders cannot be definitively determined from these data. Nevertheless, the results highlight the importance of early detection and structured radiographic follow-up to monitor lesion behavior over time.

## Figures and Tables

**Table 1:** Vertical dimensional changes in lesions according to the follow-up interval between the initial and final panoramic radiographs.

Year	*n*	Mean	SD	Med	IQR	Min.	Max.	H	*p*
1–2	27	0.237	0.236	0.200 ^b^	0.200	0.00	0.90	11.430	0.003
3–4	16	0.681	0.435	0.750 ^a^	0.680	0.00	1.60		
>5	25	0.372	0.455	0.300 ^b^	0.450	0.00	1.90		

^a,b^: different superscript letters within the same row or column indicate statistically significant differences (*p* < 0.05).

**Table 2:** Horizontal dimensional changes in lesions according to the follow-up interval between the initial and final panoramic radiographs.

Year	*n*	Mean	SD	Med	IQR	Min.	Max.	H	*p*
1–2	27	0.311	0.256	0.200 ^b^	0.300	0.00	1.00	10.379	0.006
3–4	16	0.906	0.652	0.800 ^a^	0.980	0.10	2.40		
>5	25	0.528	0.536	0.400 ^b^	0.750	0.00	1.90		

^a,b^: different superscript letters within the same row or column indicate statistically significant differences (*p* < 0.05).

**Table 3:** Comparison of vertical and horizontal dimensional change (%) between lesions with and without corticated borders on the initial panoramic radiographs.

Dimensional Increase (%)	Are Lesion’s Borders Well-Corticated	*n*	Mean	SD	Med	IQR	Min.	Max.	U-Value	*p*
Vertical plane	yes	38	31.67	63.28	13.95	28.65	0.00	366.70	291.5	<0.001
	no	30	79.72	98.79	55.00	83.63	0.00	500.0		
Horizontal plane	yes	38	35.96	46.05	17.05	44.10	0.00	183.30	273.5	<0.001
	no	30	92.01	86.96	69.05	96.63	7.70	325.00		

**Table 4:** Overall agreement between initial and second panoramic radiographs in the assessment of lesion features.

Questions	Overall κ Value	Strength of Agreement
1 What is the lesion’s shape?	0.855	Almost Perfect
2 Are its borders well-defined	0.338	Fair
3 Are its borders well-corticated in terms of thickness?	0.243	Fair
4 Are its borders continuously corticated?	0.511	Moderate
5 The lesion’s internal contents are mostly radiolucent/≤Soft tissue density, Mixed or Radiopaque/≥Bone density	1.000	Almost Perfect
6 Is the lesion multilocular?	0.656	Substantial
7 Does it appear to be affecting the incisive canal or the inferior alveolar canal?	0.567	Moderate
8 Does it appear to expand the normal surrounding anatomic boundaries?	0.544	Moderate
9 Does it appear to be causing cortical thinning?	0.530	Moderate
10 Does it appear to be causing cortical destruction?	0.523	Moderate
11 Does it appear to be causing tooth displacement?	0.472	Moderate
12 Dose it appears to be causing root resorption?	0.643	Substantial

Strength of agreement is interpreted as follows: 0.01–0.20: Slight, 0.21–0.40: Fair, 0.41–0.60: Moderate, 0.61–0.80: Substantial, 0.81–1.00: Almost perfect [16].

## Data Availability

The raw data supporting the conclusions of this article will be made available by the authors on request.
